# Laparoscopic approach for emergency colon resection: a retrospective propensity score-matched study

**DOI:** 10.1186/s13017-026-00721-y

**Published:** 2026-08-10

**Authors:** Antti J. Lahtinen, Silja A. S. Salo, Viiko Vahtera, Marie T. Grönroos-Korhonen, Jyrki A. O. Kössi

**Affiliations:** 1 Department of Gastrointestinal Surgery, Päijät-Häme Central Hospital, Lahti, Finland; 2 Department of Orthopedics, Tampere University Hospital and Tampere University, Tampere, Finland; 3 Department of Surgery, Helsinki University Hospital, Helsinki, Finland

**Keywords:** Laparoscopy, Emergency surgery, Colon, Survival, Tumor

## Abstract

**Background:**

Despite its routine use in elective procedures, the role of laparoscopy in emergency colorectal surgery remains debated due to limited evidence. This study compared laparoscopic and open surgery regarding complications, mortality, and hospital stay following emergency colon operations.

**Methods:**

This retrospective study included adult patients treated at Päijät–Häme Central Hospital between 2010 and 2020 who underwent emergency colon resection. Propensity score matching was used to adjust for comorbidities, prior ICU admission, surgical indication, and contamination between laparoscopic and open surgery groups.

**Results:**

All together 484 patients had an emergency colorectal resection of which 85 (17.6%) were carried out laparoscopically, 31 (6.4%) were converted and 377 (76.0%) were open. In the whole cohort, Comprehensive complication index was lower among laparoscopy patients than open surgery patients if intraoperative conversion to open surgery was avoided (25.8 vs. 34.9, *p* = 0.012). The subgroup analysis also showed lower CCI after laparoscopic resection for patients presented with obstruction (21.3 vs. 29.7, *p* = 0.046) or colorectal cancer (21.1 vs. 29.4, *p* = 0.020) A multivariable regression analysis also showed that starting the procedure laparoscopically predicted lower complication rates. Patients who had a laparoscopic resection also had shorter hospital stay (9.0 vs. 12.9 days, *p* < 0.001). Patients with open surgery had significantly more wound complications (10.3% vs. 1.2%, *p* = 0.010). Open surgery was also an independent risk factor for higher CCI (*p* = 0.008) and the need for intensive care (*p* = 0.027). Mortality was similar regardless of the approach in short term (30 days, *p* = 0.810; 90 days, *p* = 0.354) and in long-term survival (*p* = 0.466).

**Conclusions:**

The laparoscopic technique in colorectal emergencies may offer similar benefits as in elective surgery, with a lower burden of total complications, less wound complications and shorter hospital stay. Laparoscopy was not associated with any unexpected harmful events.

**Supplementary Information:**

The online version contains supplementary material available at https://doi.org/10.1186/s13017-026-00721-y.

## Introduction

The laparoscopic approach to elective colon surgery has become the gold standard over the last decade, offering benefits including shorter hospital stay, less pain, and improved recovery [1–2]. Despite initial concerns regarding oncological outcomes, both long-term and disease-free survival have also been shown to be comparable [1, 3] or even superior [4] to outcomes following the open approach.

However, emergency colon resections are still commonly performed via open surgery [5–6]. The emergency context itself presents distinct challenges: the patient’s condition typically demands a rapid response to acute underlying health problems, some of which are associated with substantial mortality and postoperative morbidity [5–6]. In addition to longer operating times and a steeper learning curve, these factors may lead surgeons to favor traditional open laparotomy. Some earlier studies have reported that emergency laparoscopic colorectal surgery may result in superior [7–12], or at least comparable [13–14], outcomes to open surgery; however, these studies are often limited by small sample size or significant heterogeneity among patient groups. A recent retrospective, registry-based study comparing open and laparoscopic approaches reported a lower rate of postoperative complications in patients undergoing laparoscopy [8]. The number of high-quality studies remains limited, and no randomized controlled trials have been published.

The objective of this study was to investigate whether patients benefit from laparoscopy in emergency colon resection, focusing on short-term outcomes and long-term mortality, using a large propensity score-matched patient cohort for analysis.

## Methods

### Patients and data collection

All adult patients (> 18 years) who underwent emergency colonic resection between January 1, 2010 and December 31, 2020 at Päijät–Häme Central Hospital were included. Patients were identified from electronic patient records using the Nordic Medico-Statistical Committee Surgical Procedural codes for colon resection (JFB20–JFB99), along with the separate classification code (ZXD05) indicating emergency surgery (conducted within 48 h of the diagnosis). Patients with polytrauma, abdominal aortic aneurysm rupture, acute pancreatitis, or secondary/corrective surgery were excluded. The initial data was then manually reviewed by the authors using individual medical records. Duplicates and cases involving procedures inconsistent with the initial procedure code were subsequently excluded. All the surgical procedures were done by a consultant/specialist-level gastrointestinal surgeon.

### Propensity score matching

To minimize selection bias, a propensity-score matching (PSM) analysis was performed. Baseline data were collected from medical records and following variables were included for adjustment: age, BMI, admission to the intensive care unit (ICU) or high dependency unit (HDU), reason for surgery, indication for emergency surgery (perforation, obstruction or other), and comorbidities listed in the Charlson Comorbidity Index. These included the American Society of Anesthesiologists (ASA) score, previous cerebrovascular accident or transient ischemic attack, severe heart disease (previous myocardial infarction or cardiac insufficiency), peripheral vascular disease, dementia, diabetes mellitus, chronic liver disease, chronic kidney disease, previously diagnosed cancer within 5 years, and the use of anticoagulants and oral corticosteroids [15]. Regarding the surgical procedure, data were also collected on the underlying pathology (malignant or benign), the severity of surgical site contamination, type of colectomy performed, stoma creation rate and possible type, anastomosis rate and in case of conversion from laparoscopy into open surgery, a specific reason.

**Fig. 1:**
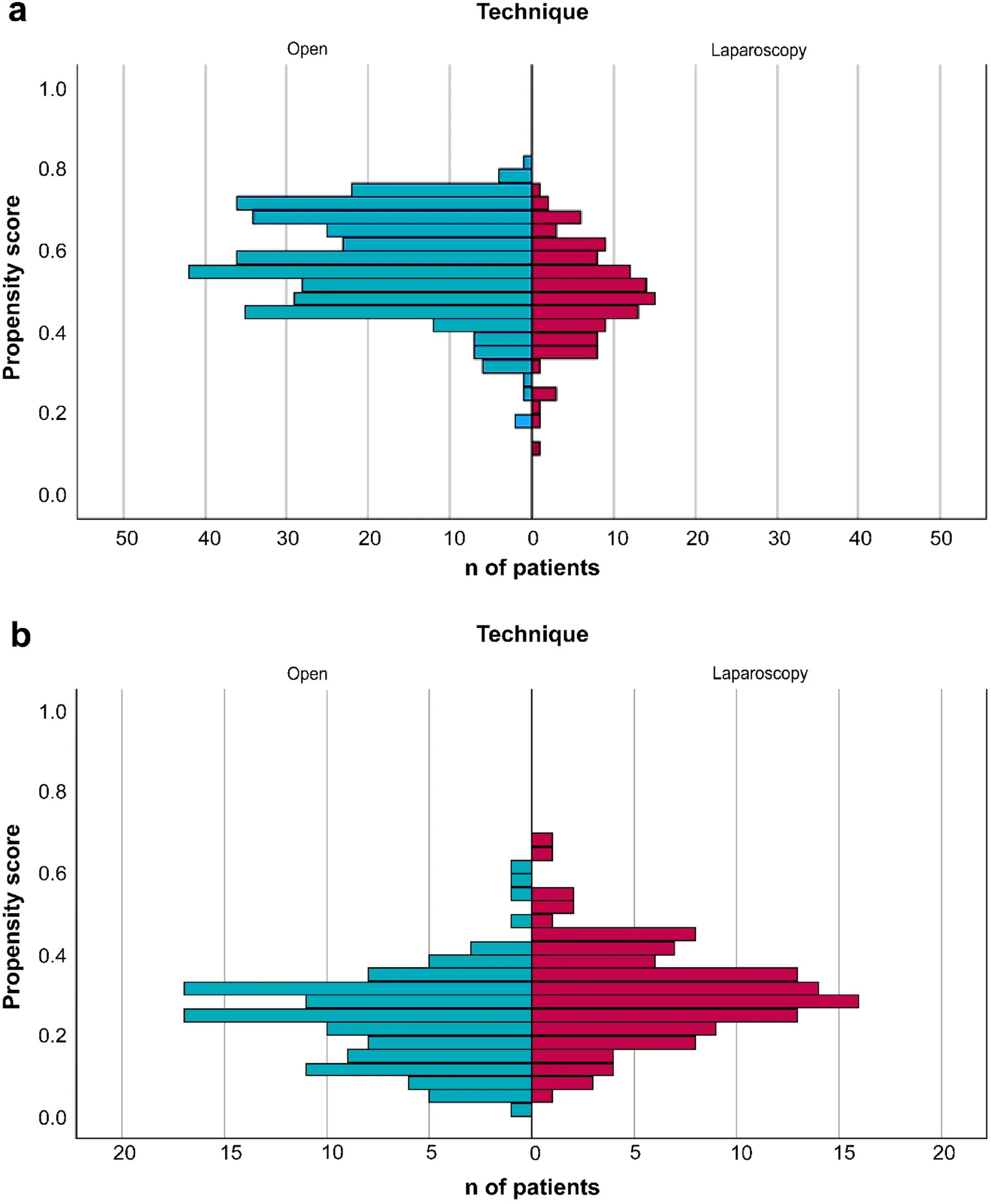
Mirrored histogram showing the distribution of propensity score before **a** and after **b** propensity score matching (PSM) analysis

For the PSM analysis, patients were categorized according to surgical approach: laparoscopic resection (including conversions, based on the “intention-to-treat [ITT] principle”) or open resection. The PSM algorithm in SPSS (version 29.0.1.0) was used. As fewer patients underwent laparoscopic resection, patients in the open group were selected as controls. The match tolerance was set at 0.2 standard deviations of the logit of the propensity score. Matching was conducted 1:1 without replacement, and all patients who underwent minimally invasively surgery were included in the cohort. The standardized mean difference (SMD), which quantifies the difference between two group means for one or more variables, was used to assess covariate balance before and after PSM [16]. To evaluate the effectiveness of the matching process, a mirrored histogram was generated to visually depict the distribution of SMD values and the balance achieved in covariate distributions between the matched groups (Fig. 1, Supplementary Table 1).

### Outcomes of interest

The primary objective was to compare postoperative recovery between laparoscopic and open emergency colon resection by evaluating morbidity, length of hospital stay, and mortality. To better analyze the effect of conversion, the surgical approach was compared using two different variables: laparoscopic ITT and laparoscopic as-treated (AT). The laparoscopy ITT group comprised patients who underwent a laparoscopic resection or a procedure that was initially started laparoscopically but converted to open surgery. The procedure was considered conversion surgery only for cases where the surgical procedure was actually performed laparoscopically at the start (thus, excluding the cases where the conversion was done immediately after the insertion of the laparoscope) and, for a specific reason stated by a surgeon, were converted into open approach. The reasons for conversion are presented in supplementary Table 2. The open group included patients who underwent an open procedure. The laparoscopy AT classification comprised laparoscopy, conversion, and open techniques as separate categorical variables. Postoperative complications, including their type and severity within 30 days of surgery, were collected from medical records in accordance with Clavien–Dindo grading system [17]. Complications requiring ICU or HDU care were classified as grade IVb and IVa, respectively. The ICU is a department in the hospital that provides care for patients with severe, life-threatening conditions, managed primarily by anesthesiologists and offering the highest nurse-to-patient ratio. The HDU serves as an intermediate ward for patients requiring close monitoring and care not available in a regular ward.

To describe the cumulative burden of complications, the Comprehensive Complication Index (CCI) was calculated using the formula: CCI = √(wC + wC2+….wCx)/2, where C represents each complication and w denotes a value based on severity, according to the Clavien-Dindo classification system, yielding a score from 0 to 100 [17]. In cases of death, the maximum score (100) was applied. Details of postoperative bowel function were also extracted from the medical records.

At the time of discharge, patients were either sent home or transferred to a primary healthcare center ward if further hospitalization or rehabilitation was deemed necessary.

Postoperative survival was assessed for a minimum of 5 years postoperatively, and the cause of death was recorded where applicable. For patients who had relocated to another hospital district, the most recent recorded contact with the healthcare system was reviewed to confirm that the patient was alive on that particular date.

A subgroup analysis on hospital stay, admission to ICU/HDU, total CCI and mortality was conducted for specific surgical indications (obstruction, perforation, other indications), presence of colorectal cancer and for the type of hemicolectomy (left, right).

### Statistical analysis

Statistical analyses were performed using IBM SPSS statistics for windows (version 29.0.1.0; IBM ). Categorical variables were compared using the chi-square test or Fisher’s exact test, and continuous variables using the Mann–Whitney U-test. The comparison was performed for the whole study population and for patient subgroups based on emergency surgery indication (obstruction vs. perforation vs. other indications), presence of colorectal cancer, and type of resection (left vs. right). *P*-value < 0.05 was considered statistically significant. Multivariable regression analysis was performed to assess total CCI and 30- and 90-day postoperative mortality, and conversion risk for laparoscopy patients. All baseline and operative characteristics that showed statistical significance in univariable analysis were included in the multivariable analysis. Kaplan–Meier estimator was used for the long-term survival.

### Ethics

The study was approved by the Institutional Review Board of Päijät–Häme Central Hospital. Ethical committee approval was not required, as the study was a retrospective review of patient records.

## Results

Following screening, the dataset included 484 patients, of whom 85 underwent laparoscopic surgery, 31 required conversion, and 368 had open procedures. The final matched cohort comprised 116 patients who underwent minimally invasive surgery (including 31 conversions) and 116 who underwent open surgery.

After PSM, baseline characteristics were similar between the two groups (laparoscopy ITT and open), except for dementia (*p* = 0.039, Table 1). Significant differences were also observed in the number of prior operations (*p* = 0.032), the degree of surgical site contamination (*p* = 0.014) and the type of resection (*p* = 0.007, Table 1). Similarly, when comparing all three groups separately (laparoscopy, conversion, and open surgery), no significant differences were found, apart from dementia (*p* = 0.041), surgical site contamination (*p* = 0.028) and type of resection (*p* = 0.047, Table 2). The mirrored histogram illustrates that the distribution of baseline characteristics between the groups was more balanced following PSM (Fig. 1). The detailed SMDs for specific variables are presented in the supplementary material (Supplementary Table 1). The most common reasons for conversion were technical difficulties (13 cases) and insufficient working space (12 cases, Supplementary Table 2). A multivariate analysis found that chronic liver disease was a predictor of conversion (*p* = 0.027, Supplementary Table 2).

**Table 1:** Baseline characteristics

		Open	Laparoscopy ITT	
		*n* = 116	*n* = 116	*p*-value
*Demographic and medical characteristics*				
Age^a^		68.3 (15.31)	69.3 (14.29)	*p* = 0.767
Body mass index^a^		25.5 (4.30)	25.5 (4.66)	*p* = 0.682
Charlson Comorbidity Index		4.6 (2.61)	4.9 (2.97)	*p* = 0.677
Sex				*p* = 0.692
Female		66 (56.9%)	62 (53.4%)	
Male		50 (43.1%)	74 (63.8%)	
ASA grade				*p* = 0.140
I		1 (0.9%)	4 (3.4%)	
II		34 (29.3%)	32 (27.6%)	
III		51 (44.0%)	63 (54.3%)	
IV		27 (23.3%)	16 (13.8%)	
V		3 (2.6%)	1 (0.9%)	
Cancer within 5 years				*p* = 0.581
Local		37 (31.9%)	39 (33.6%)	
Metastatic		15 (12.9%)	20 (17.2%)	
Cerebrovascular accident or TIA		14 (12.1%)	13 (11.2%)	*p* = 1.000
Severe heart disease		22 (19.0%)	20 (17.2%)	*p* = 0.865
Peripheral vascular disease		4 (3.4%)	7 (6.0%)	*p* = 0.539
Dementia		19 (16.4%)	8 (6.9%)	*p* = 0.039
Diabetes mellitus		11 (9.5%)	15 (12.9%)	*p* = 0.533
Chronic liver disease		3 (2.6%)	2 (1.7%)	*p* = 1.000
Chronic kidney disease		6 (5.2%)	4 (3.4%)	*p* = 0.748
Use of anticoagulants		14 (12.1%)	15 (12.9%)	*p* = 1.000
Use of oral corticosteroids		6 (5.2%)	5 (4.3%)	*p* = 1.000
Admittance to ICU or HDU before surgery		5 (4.3%)	8 (6.9%)	*p* = 0.570
*Operation characteristics*				
Operation time (min) ^a^		145.36 (61.79)	154.65 (51.51)	*p* = 0.052
Prior operations^a^		0.78 (1.08)	0.42 (0.742)	*p* = 0.032
Reason for surgery				*p* = 0.749
Malignant tumour (CRC)		42 (36.2%)	48 (41.4%)	
Malignant tumor (not CRC)		3 (2.6%)	3 (2.6%)	
Benign obstruction		28 (24.1%)	19 (16.4%)	
Ischemia		4 (3.4%)	6 (5.2%)	
Diverticulitis		33 (28.4%)	29 (25.0%)	
Colitis / IBD		2 (1.7%)	4 (3.4%)	
Other		4 (3.4%)	7 (6.0%)	
Emergency surgery indication				*p* = 0.350
Obstruction		55 (47.4%)	57 (49.1%)	
Perforation		40 (34.5%)	29 (25.0%)	
Other		21 (18.1%)	30 (25.9%)	
Surgical site contamination				*p* = 0.014
None		64 (55.2%)	80 (69.0%)	
Local		25 (21.6%)	25 (21.6%)	
Peritonitis		27 (23.3%)	11 (9.5%)	
Type of colectomy				*p* = 0.007
Right sided resection		37 (31.9%)	59 (50.9%)	
Left sided resection		61 (52.6%)	50 (43.1%)	
Rectal resection		2 (1.7%)	1 (0.9%)	
Other^b^		16 (13.8%)	6 (5.2%)	
Primary Anastomosis		84 (72.4%)	95 (81.9%)	*p* = 0.360
Stoma				*p* = 0.228
None		71 (61.2%)	83 (71.6%)	
Protective stoma (colon)		5 (4.3%)	8 (6.9%)	
Protective stoma (ileum)		8 (6.9%)	4 (3.4%)	
End colostomy		24 (20.7%)	14 (12.1%)	
End ileostomy		8 (6.9%)	7 (6.0%)	

Laparoscopy group includes conversions

TIA, Transient ischemic attack; IBD, Inflammatory bowel disease; ICU, Intensive care unit; HDU, High dependency unit; CRC, Colorectal cancer; ITT, Intention-to-treat

^a^Values are: mean (SD)

^b^Open: 1 case of transversum resection, 15 cases of other type of colectomies. Laparoscopy ITT: 1 case of transversum resection, 5 cases of other type of colectomies

**Table 2:** Baseline characteristics

	Open	Laparoscopy AT	Conversion	*p*-value
	n = 116	n = 85	n = 31	
Demographic and medical characteristics				
Age^a^	68.3 (15.31)	69.6 (13.09)	68.5 (17.57)	*p* = 0.900
Body mass index^a^	25.5 (4.30)	25.4 (4.91)	25.8 (3.91)	*p* = 0.574
Charlson Comorbidity Index	4.6 (2.61)	5.0 (2.85)	4.6 (3.28)	*p* = 0.622
Sex				*p* = 0.743
Female	66 (56.9%)	44 (51.8%)	18 (58.1%)	
Male	50 (43.1%)	41 (48.2%)	13 (41.9%)	
ASA grade				*p* = 0.376
I	1 (0.9%)	2 (2.4%)	2 (6.5%)	
II	34 (29.3%)	24 (28.2%)	8 (25.8%)	
III	51 (44.0%)	46 (54.1%)	17 (54.8%)	
IV	27 (23.3%)	12 (14.1%)	4 (12.9%)	
V	3 (2.6%)	1 (1.2%)	0	
Cancer within 5 years				*p* = 0.627
Local	37 (31.9%)	31 (36.5%)	8 (25.8%)	
Metastatic	15 (12.9%)	15 (17.6%)	5 (16.1%)	
Cerebrovascular accident or TIA	14 (12.1%)	12 (14.1%)	1 (3.2%)	*p* = 0.270
Severe heart disease	22 (19.0%)	15 (17.6%)	5 (16.1%)	*p* = 0.917
Peripheral vascular disease	4 (3.4%)	5 (5.9%	2 (6.5%)	*p* = 0.622
Dementia	19 (16.4%)	4 (4.7%)	4 (12.9%)	*p* = 0.041
Diabetes mellitus	11 (9.5%)	13 (15.3%)	2 (6.5%)	*p* = 0.299
Chronic liver disease	3 (2.6%)	0	2 (6.5%)	*p* = 0.066
Chronic kidney disease	6 (5.2%)	3 (3.5%)	1 (3.2%)	*p* = 0.902
Use of anticoagulants	14 (12.1%)	12 (14.1%)	3 (9.7%)	*p* = 0.793
Use of oral corticosteroids	6 (5.2%)	4 (4.7%)	1 (3.2%)	*p* = 1.000
Admittance to ICU or HDU before surgery	5 (4.3%)	6 (7.1%)	2 (6.5%)	*p* = 0.663
*Operation characteristics*				
Operation time (min)^a^	145.36 (61.79)	150.60 (49.92)	166.41 (55.08)	*p* = 0.076
Prior operations^a^	0.78 (1.08)	0.43 (0.75)	0.41 (0.73)	*p* = 0.097
Reason for surgery				*p* = 0.192
Malignant tumour (CRC)	42 (36.2%)	41 (48.2%)	7 (22.6%)	
Malignant tumor (not CRC)	3 (2.6%)	1 (1.2%)	2 (6.5%)	
Benign obstruction	28 (24.1%)	14 (16.5%)	5 (16.1%)	
Ischemia	4 (3.4%)	5 (5.9%)	1 (3.2%)	
Diverticulitis	33 (28.4%)	16 (18.8%)	13 (41.9%)	
Colitis / IBD	2 (1.7%)	2 (2.4%)	2 (6.5%)	
Other	4 (3.4%)	6 (7.1%)	1 (3.2%)	
Emergency surgery indication				*p* = 0.156
Obstruction	55 (47.4%)	45 (52.9%)	12 (38.7%)	
Perforation	40 (34.5%)	16 (18.8%)	13 (41.9%)	
Other	21 (18.1%)	24 (28.2%)	6 (19.4%)	
Surgical site contamination				*p* = 0.028
None	64 (55.2%)	62 (72.9%)	18 (58.1%)	
Local	25 (21.6%)	17 (20.0%)	8 (25.8%)	
Peritonitis	27 (23.3%)	6 (7.1%)	5 (16.1%)	
Type of colectomy				*p* = 0.047
Right sided resection	37 (31.9%)	45 (52.9%)	14 (45.2%)	
Left sided resection	61 (52.6%)	36 (42.4%)	14 (45.2%)	
Rectal resection	2 (1.7%)	1 (1.2%)	0	
Other	16 (13.8%)	3 (3.5%)	3 (9.7%)	
Stoma				*p* = 0.222
None	71 (61.2%)	62 (72.9%)	21 (67.7%)	
Protective stoma (colon)	5 (4.3%)	7 (8.2%)	1 (3.2%)	
Protective stoma (ileum)	8 (6.9%)	4 (4.7%)	0	
End colostomy	24 (20.7%)	7 (8.2%)	7 (22.6%)	
End ileostomy	8 (6.9%)	5 (5.9%)	2 (6.5%)	
Anastomosis	84 (72.4%)	73 (85.9%)	22 (71.0%)	*p* = 0.309

Conversion as a separate group

TIA, Transient ischemic attack; IBD, Inflammatory bowel disease; ICU, Intensive care unit; HDU, High dependency unit; CRC, Colorectal cancer; AT, As-treated

^a^Values are: mean (SD)

^b^Open: 1 case of transversum resection, 15 cases of other type of colectomies. Laparoscopy: 3 cases of other type of colectomies. Conversion: 1 case of transversum resection, 2 cases of other type of colectomies

Postoperative complications are presented in Tables 3 and 4. Surgical wound complications were significantly less frequent in the laparoscopy group in both the ITT and AT analyses compared with the open surgery group (*p* = 0.010 and *p* = 0.023, respectively). Total CCI was lowest among patients who underwent laparoscopy without conversion (mean 25.8, *p* = 0.012), compared with those who required conversion (mean 43.2) or underwent open surgery (mean 34.9, Table 4). However, no differences were observed when patients in the laparoscopy and conversion groups were combined and compared with the open surgery group (*p* = 0.256, Table 3). The results remained consistent when patients who died within the first 30 days postoperatively were excluded. No differences were observed in the restitution of postoperative bowel function between the different surgery approaches (*p* = 0.411, Table 3).

**Table 3:** Postoperative complications within 30 days and total Comprehensive Complication Index

				Open		Laparoscopy ITT		
				*n* = 116		*n* = 116		*p*-value
*Surgical complications*								
Delayed bowel motility^a^				25 (21.6%)		30 (25.9%)		*p* = 0.537
Infection								
Superficial				6 (5.2%)		6 (5.2%)		*p* = 1.000
Deep (abscess)				5 (4.3%)		9 (7.8%)		*p* = 0.409
Anastomotic leakage				6 (5.2%)		9 (7.8%)		*p* = 0.595
Anemia				9 (7.8%)		8 (6.9%)		*p* = 1.000
Wound dehiscence^b^				12 (10.3%)		2 (1.7%)		*p* = 0.010
Fascial dehiscence				6 (5.2%)		1 (0.9%)		*p* = 0.119
Requiring reoperation								
Pain				9 (7.8%)		13 (11.2%)		*p* = 0.502
Stoma complication				2 (1.7%)		4 (3.4%)		*p* = 0.683
Reoperation for other reason^c^				0		1 (0.9%)		*p* = 1.000
*Medical complications*								
Cardiovascular complications								
Pulmonary embolism				5 (4.3%)		2 (1.7%)		*p* = 0.446
Deep vein thrombosis				0		1 (0.9%)		*p* = 1.000
Other cardiac complication				21 (18.1%)		26 (22.4%)		*p* = 0.514
Respiratory complications				11 (9.5%)		12 (10.3%)		*p* = 1.000
Neurological complications								
Cerebrovascular accident or hemorrhage				1 (0.9%)		0		*p* = 1.000
Other neurological complications				0		1 (0.9%)		*p* = 1.000
GI-complications not related to surgery								
Diarrhea				4 (3,4%)		4 (3.4%)		*p* = 1.000
Reflux				7 (6.0%)		3 (2.6%)		*p* = 0.333
Urinary tract complications^d^				1 (1.4%)		8 (6.9%)		*p* = 0.100
Delirium				11 (9.5%)		10 (8.6%)		*p* = 1.000
Infection not related to surgery				18 (15.5%)		15 (12.9%)		*p* = 0.707
Electrolyte imbalance				10 (8.6%)		13 (11.2%)		*p* = 0.661
Allergic reaction				1 (0.9%)		1 (0.9%)		*p* = 1.000
				Mean (SD)		Mean (SD)		
CCI 30 days postoperatively				34.9 (29.64)		30.5 (28.79)		*p* = 0.256
CCI (deceased patients excluded)				28.8 (22.81)		27.5 (25.69)		*p* = 0.532
Postoperative bowel function, days				4.0 (2.94)		3.8 (2.98)		*p* = 0.411

Laparoscopy group includes conversions

SD, Standard deviation; GI, Gastrointestinal; CCI, Comprehensive complication index; ITT, Intention-to-treat

^a^Delayed bowel function requiring medical intervention, insertion or re-insertion of nasogastric tube,

or major operation

^b^Partial or total separation of wound edges or fascia

^c^One postoperative case of intestinal necrosis

^d^No cases of urinary tract injuries due to surgery

**Table 4:** Postoperative complications within 30 days and total Comprehensive Complication Index

	Open	Laparoscopy AT	Conversion	
	n = 116	n = 85	n = 31	*p*-value
*Surgical complications*				
Delayed bowel motilityᵃ	25 (21.6%)	21 (24.7%)	9 (29.0%)	*p* = 0.666
Infection				
Superficial	6 (5.2%)	4 (4.7%)	2 (6.5%)	*p* = 0.844
Deep (abscess)	5 (4.3%)	5 (5.9%)	4 (12.9%)	*p* = 0.199
Anastomotic leakage	6 (5.2%)	6 (6.3%)	3 (9.7%)	*p* = 0.667
Anemia	9 (7.8%)	6 (7.1%)	2 (6.5%)	*p* = 1.000
Wound dehiscenceᵇ	12 (10.3%)	1 (1.2%)	1 (3.2%)	*p* = 0.023
Fascial dehiscence	6 (5.2%)	0	1 (3.2%)	*p*=0.067
requiring reoperation				
Pain	9 (7.8%)	9 (10.6%)	4 (12.9%)	*p* = 0.640
Stoma complication	2 (1.7%)	2 (2.4%)	2 (6.5%)	*p* = 0.285
Reoperation for other reasonᶜ	0	1 (1.2%)	0	*p* = 0.500
*Medical complications*				
Cardiovascular complications				
Pulmonary embolism	5 (4.3%)	1 (1.2%)	1 (3.2%)	*p*=0.487
Deep vein thrombosis	0	1 (1.2%)	0	*p*=0.500
Other cardiac complication	21 (18.1%)	16 (18.8%)	10 (32.3%)	*p*=0.215
Respiratory complications	11 (9.5%)	7 (8.2%)	5 (16.1%)	*p* = 0.444
Neurological complications				
Cerebrovascular accident	1 (0.9%)	0	0	*p* = 1.000
or hemorrhage				
Other neurological	0	1 (1.2%)	0	*p* = 0.500
complications				
GI-complications not				
related to surgery				
Diarrhea	4 (3,4%)	2 (2.4%)	2 (6.5%)	*p* = 0.635
Reflux	7 (6.0%)	2 (2.4%)	1 (3.2%)	*p* = 0.493
Urinary tract complicationsᵈ	1 (1.4%)	5 (6.0%)	3 (9.7%)	*p* = 0.110
Delirium	11 (9.5%)	6 (7.1%)	4 (12.9%)	*p* = 0.627
Infection not related to surgery	18 (15.5%)	10 (11.8%)	5 (16.1%)	*p* = 0.755
Electrolyte imbalance	10 (8.6%)	10 (11.8%)	3 (9.7%)	*p* = 0.791
Allergic reaction	1 (0.9%)	1 (1.2%)	0	*p* = 1.000
	Mean (SD)	Mean (SD)	Mean (SD)	
CCI 30 days postoperatively	34.9 (29.64)	25.8 (26.52)	43.2 (31.32)	*p* = 0.012^e^
CCI (deceased patients excluded)	28.8 (22.82)	23.1 (22.77)	39.9 (29.61)	*p* = 0.019^f^
Postoperative bowel function, days	4.0 (2.94)	3.5 (2.37)	4.8 (4.16)	*p* = 0.062

Conversions as a separate group

SD, Standard deviation; GI, Gastrointestinal; CCI, Comprehensive complication index; AT, As-treated

^a^Delayed bowel function requiring medical intervention, insertion or re-insertion of nasogastric tube, or major operation

^b^Partial or total separation of wound edges or fascia

^c^One postoperative case of intestinal necrosis

^d^No cases of urinary tract injuries due to surgery

^e^Kruskal–Wallis test: laparoscopic vs. open, *p* = 0.033; laparoscopic vs. conversion, *p* = 0.006; open vs. conversion, *p* = 0.176

^f^Kruskal–Wallis test: laparoscopic vs. open, *p* = 0.102; laparoscopic vs. conversion, *p* = 0.006; open vs. conversion, *p* = 0.091

Discharge straight home from hospital was comparable between the laparoscopy and open surgery groups both in the ITT and AT analyses (*p* = 0.110 and *p* = 0.175, respectively, Tables 5 and 6). Similarly, there were no differences in the risk of readmission within 30 days of discharge (*p* = 0.830 and *p* = 0.912, respectively, Tables 5 and 6). Admission rates to the ICU or HDU were also similar between the two groups (*p* = 0.763, Table 5). However, when converted patients were analyzed separately, those in the laparoscopy group had the lowest rate of ICU/HDU admission (17.6%, *p* = 0.027) compared with the conversion (41.9%) and open surgery (26.7%, Table 6) groups. This difference was particularly evident in the case of ICU admission (*p* = 0.033). Mortality at 30 and 90 days postoperatively did not differ regardless of the type of operation (*p* = 0.285 and *p* = 1.000, respectively, Tables 5 and 6).

**Table 5:** Duration of postoperative hospital stay, ICU and HDU stay, discharge destination, and mortality within 90 days

		Open	Laparoscopy ITT	*p*-value
		*n* = 116	*n* = 116	
Admitted to ICU or HDU		31 (26.7%)	28 (24.1%)	*p* = 0.763
Postoperatively				
ICU		10 (8.6%)	7 (6.0%)	*p* = 0.616
HDU		25 (21.6%)	26 (2.4%)	*p* = 1.000
Discharged to^a^				*p* = 0.110
Home		61 (52.6%)	74 (64.9%)	
Other healthcare facility		55 (47.4%)	40 (35.1%)	
Returned to hospital within 30 days		11 (9.6%)	13 (11.2%)	*p* = 0.830
Mortality				
30 days		10 (8.6%)	5 (4.3%)	*p* = 0.285
90 days		15 (12.9%)	16 (13.8%)	*p* = 1.000
		Mean (SD)	Mean (SD)	
Hospital stay, days^a^		12.9 (14.02)	9.8 (7.08)	*p* = 0.002
Postoperative ICU or HDU stay, days		1.3 (3.22)	1.8 (5.95)	*p* = 0.782
ICU stay		0.5 (1.95)	0.7 (3.89)	*p* = 0.484
HDU stay		0.7 (1.91)	1.1 (2.60)	*p* = 0.762

Laparoscopy group includes conversions

ICU, Intensive care unit; HDU; High dependency unit; SD, Standard deviation; ITT, Intention-to-treat

^a^ Excluded patients who died in the hospital

**Table 6:** Duration of postoperative hospital stay, ICU and HDU stay, discharge destination, and mortality within 90 days. Conversions as a separate group

		Open	Laparoscopy AT	Conversion			*p*-value
		*n* = 116	*n* = 85	*n* = 31			
Admitted to ICU or HDU		31 (26.7%)	15 (17.6%)	13 (41.9%)			*p* = 0.027
Postoperatively							
ICU		10 (8.6%)	2 (2.4%)	5 (16.1%)			*p* = 0.033
HDU		25 (21.6%)	15 (17.6%)	11 (35.5%)			*p* = 0.130
Discharged to^a^							*p* = 0.175
Home		61 (52.6%)	56 (65.9%)	18 (58.1%)			
Other healthcare facility		55 (47.4%)	27 (34.1%)	13 (41.9%)			
Returned to hospital within 30 days		11 (9.6%)	10 (11.8%)	3 (9.7%)			*p* = 0.912
Mortality							
30 days		10 (8.6%)	3 (3.5%)	2 (6.5%)			*p* = 0.373
90 days		15 (12.9%)	9 (10.6%)	7 (22.6%)			*p* = 0.256
		Mean (SD)	Mean (SD)	Mean (SD)			
Hospital stay, days^a^		12.9 (14.02)	9.0 (5.38)	12.2 (10.47)			*p* < 0.001
Postoperative ICU or HDU stay, days		1.3 (3.22)	1.1 (3.47)	4.0 (10.13)			*p* = 0.013
ICU stay		0.5 (1.95)	0.3 (2.27)	2.0 (6.68)			*p* = 0.032
HDU stay		0.7 (1.91)	0.8 (1.84)	2.0 (4.02)			*p* = 0.059

ICU, Intensive care unit; HDU, High dependency unit; SD, Standard deviation; AT, As-treated

^a^ Excluded patients who died in the hospital

Total length of hospital stay was significantly shorter in the laparoscopy ITT group compared with the open surgery group (*p* = 0.002, Table 5). The shortest hospital stay was observed in patients who underwent laparoscopy without conversion (mean 9.0 days, *p* < 0.001, Table 6). No differences were observed between the laparoscopy ITT and open surgery groups regarding the duration of treatment in the ICU or HDU (*p* = 0.782, Table 5), although patients who underwent successful laparoscopy without conversion had the shortest overall stay (mean 1.1 days, *p* = 0.013, Table 6). This difference was particularly evident in ICU stay (*p* = 0.032), but not in HDU stay (*p* = 0.059).

In the subgroup analysis, patients presented with obstruction or CRC had a significantly shorter hospital stay (8.5 vs. 11.7 days, *p* = 0.013 and 8.5 vs. 14.5 days, *p* = 0.013 respectively) and lower CCI (21.3 vs. 29.7, *p* = 0.046 and 21.1 vs. 29.4, *p* = 0.020, respectively) after laparoscopy compared to open surgery in both ITT and as treated analyses (Table 7). No other significant differences were found between laparoscopy ITT and open approach (Table 7). Intraoperative conversion from laparoscopy to open approach resulted in worst outcomes in several subgroups (Table 7). Risk factors for 30- and 90-day mortality in multivariable analysis are presented in Table 8. ASA classification and severe heart disease were identified as independent risk factors for 30-day mortality (*p* = 0.009 and *p* = 0.048, respectively), whereas high ASA classification (*p* = 0.008), peripheral vascular disease (*p* = 0.010), and cancer diagnosed within 5 years (*p* = 0.008) were identified as independent risk factors for 90-day mortality (Table 8). Independent risk factors for high CCI included high ASA classification (*p* = 0.004), chronic liver disease (*p* = 0.007), admission to the ICU or HDU prior to surgery (*p* = 0.003), and surgical site contamination (*p* = 0.019). Laparoscopic resection was associated with lower CCI when conversion was analyzed separately as a third group (*p* = 0.008).

Long-term survival was similar between the groups (Supplementary Table 3). No differences were observed regardless of the surgical approach, whether the conversion from laparoscopy to open was considered as a separate group (laparoscopy AT, *p* = 0.456) or part of the laparoscopy group (laparoscopy ITT, *p* = 0.466).

## Discussion

Key findings of the present study were that the laparoscopic technique was feasible in the emergency setting, with an acceptable conversion rate, and was not associated with unwanted adverse events. Furthermore, laparoscopy was associated with a lower cumulative burden of postoperative complications, reflected by reduced CCI scores, especially in patients presented with obstruction or colorectal cancer.

Complication rates in specific complication types were similar between the minimally invasive and open surgery groups, except for wound-related problems, which, as expected, were more common following open surgery. The total amount and severity of complications in both direct comparison and in multivariable analysis was lower in the laparoscopy group compared with the open group, but only when conversion was avoided. Conversion from laparoscopy predicted the worst outcome. Converted patients were more likely to require ICU treatment and had higher CCI scores, reflecting an increased burden of postoperative complications. These findings may indicate that some patients are less suitable for a laparoscopic approach. This notion is further reinforced by the observation that the most frequent reasons for conversion were inadequate operative exposure, limited working space, and other intraoperative technical challenges. However, as mortality rates were similar between the groups, the higher complication burden among converted patients may simply reflect the inherent complexity of these cases rather than the choice of operative approach itself. Contrastingly, our subgroup analysis showed that the complication burden was lower in laparoscopy group regardless of conversion, but only in patients diagnosed with bowel obstruction (without perforation) or colorectal carcinoma. Patients with any other indication for surgery showed similar complications rate for both techniques. Previous studies have reported conflicting results concerning postoperative complications in emergency colorectal procedures. A recent retrospective registry-based study by Chang et al. [8] which employed PSM and focused on colorectal cancer emergencies, reported findings broadly consistent with those of the present study. It observed a higher incidence of specific complications, such as ileus and pulmonary issues, among patients undergoing open surgery compared with those receiving laparoscopic procedures. In contrast, other retrospective studies [7, 9] found no differences in postoperative complications between the open and laparoscopy groups. These discrepancies may be attributed to differences in the reporting of complications, as earlier studies either used dichotomic (yes/no) reporting of specific complication types or reported only the total number of complications. Interestingly, no previous studies have found differences in postoperative wound complications, despite this being a well-recognized advantage of elective laparoscopic surgery [5]. In general, the variations between earlier studies and the present one are most likely explained by differences in the methods used to collect and report postoperative complications.

Postoperative bowel motility recovered slightly faster among patients in the laparoscopy AT group than those undergoing open surgery, although the difference was not significant. Similarly, delayed bowel motility was the most common adverse effect requiring medical intervention across all patient groups, with no significant differences observed between surgical approaches. This may be explained by the fact that recovery of postoperative bowel motility in emergency patients is more influenced by the prevailing disease (colonic perforation or obstruction) than by the surgical approach itself. Previous studies have either reported inconclusive findings [14, 10] or have favored laparoscopy over open surgery in the emergency setting regarding the postoperative risk of ileus [8, 9, 18–21]. A recent systematic review and meta-analysis summarized these results and reported a lower risk of ileus among laparoscopically treated patients [18]. However, the definition of ileus was poorly defined in the included studies [8–10, 14, 19–21] and, unlike in the present study, postoperative time to bowel function was reported in only one study [10], making comparison with the current findings challenging.

Hospital stay was shorter among patients in both the laparoscopy ITT and laparoscopy AT groups, although this was primarily observed in those who underwent laparoscopy without conversion. Discharge destination and the rate of hospital readmission within one month postoperatively were similar regardless of surgical approach. However, admission to the ICU or HDU and prolonged treatment in these units were more common following open or conversion surgery, likely reflecting a higher severity of postoperative complications in these groups. A subgroup analysis found that the shorter hospital stay among laparoscopy ITT patients was especially true for patients with obstruction without perforation and for patients diagnosed with colorectal cancer. Previous studies have reported slightly shorter hospital stays among patients undergoing laparoscopic surgery [8, 9, 12], which aligns with the present findings. Only one earlier study, by Alselaim et al. [7] collected data on postoperative intensive care, reporting that laparoscopic surgery was associated with a shorter length of stay in the ICU, consistent with the results observed in this study.

Both 30-day and 90-day mortality rates and long-term (at least 5 years follow-up) were similar among the patients regardless of the surgical approach. A high ASA classification grade, heart and vascular diseases, and a prior cancer diagnosis were independent predictors of increased mortality. Previous studies have reported a correlation between the laparoscopic approach and reduced short-term mortality [8, 12], whereas others found no such correlation [7, 9, 13]. Estimating the precise association between mortality and surgical technique remains challenging, as comorbidities and the emergency setting may influence both the choice of surgical approach and postoperative outcomes. Vallance et al. [12] found that poor preoperative physical status and advanced stage of malignant tumor strongly reduced the likelihood of undergoing laparoscopic surgery in colorectal cases. Similarly, Alselaim et al. [7] reported a significant difference in 30-day postoperative mortality between surgical approaches, 3.4% following laparoscopy versus 14.3% after open surgery, but this difference disappeared after adjusting for preoperative characteristics such as comorbidities, underlying disease, and ASA classification. In contrast, Chang et al. [8] found that open surgery was associated with higher mortality irrespective of preoperative comorbidities, potentially due to an increased incidence of postoperative pulmonary complications.

**Table 7:** Subgroup analysis of postoperative hospital stay, mortality and comprehensive complication index

		Open	Laparoscopy ITT*	Laparoscopy AT**		Lap ITT*	Lap AT**
				Laparoscopy	Conversion		
						*p*-value	*p*-value
*Obstruction as emergency surgery indication*		*n* = 55	*n* = 57	*n* = 45	*n* = 12		
Admitted to ICU or HDU postoperatively		13 (23.6%)	10 (17.5%)	5 (11.1%)	5 (41.7%)	*p* = 0.425	*p* = 0.048
Mortality 30 days		2 (3.6%)	3 (5.3%)	1 (2.2%)	2 (16.7%)	*p* = 1.000	*p* = 0.090
Mortality 90 days		4 (7.3%)	8 (14.0%)	4 (8.9%)	4 (33.3%)	*p* = 0.247	*p* = 0.027
Hospital stay (days) ^a^		11.7 (15.94)	8.5 (5.64)	8.2 (5.80)	10.2 (4.76)	*p* = 0.013	*p* = 0.003
ICU or HDU stay, days^a^		1.1 (3.21)	1.3 (4.41)	0.9 (4.17)	2.8 (5.33)	*p* = 0.515	*p* = 0.033
CCI 30 days postoperatively^a^		29.7 (21.18)	21.3 (23.91)	18.6 (21.18)	33.1 (32.14)	*p* = 0.046	*p* = 0.017
*Perforation as emergency surgery indication*		*n* = 40	*n* = 29	*n* = 16	*n* = 13		
Admitted to ICU or HDU postoperatively		12 (30.0%)	9 (31.0%)	2 (12.5%)	7 (53.8%)	*p* = 0.927	*p* = 0.055
Mortality 30 days		6 (15.0%)	1 (3.4%)	1 (6.3%)	0	*p* = 0.255	*p* = 0.251
Mortality 90 days		8 (20.0%)	6 (20.7%)	3 (18.8%)	3 (23.1%)	*p* = 0.957	*p* = 0.944
Hospital stay (days) ^a^		14.9 (3.83)	13.0 (11.05)	9.5 (3.83)	17.0 (15.00)	*p* = 0.367	*p* = 0.128
ICU or HDU stay, days^a^		1.62 (3.60)	4.3 (11.27)	0.60 (1.68)	8.6 (15.65)	*p* = 0.911	*p* = 0.048
CCI 30 days postoperatively^a^		28.5 (19.47)	38.2 (24.78)	26.5 (19.47)	51.68 (23.94)	*p* = 0.579	*p* = 0.152
*Other emergency surgery indications*		*n* = 21	*n* = 30	*n* = 24	*n* = 6		
Admitted to ICU or HDU postoperatively		6 (28.6%)	9 (30.0%)	8 (33.3%)	1 (16.7%)	*p* = 0.912	*p* = 0.721
Mortality 30 days		2 (9.5%)	1 (3.3%)	1 (4.2%)	0	*p* = 0.561	*p* = 0.605
Mortality 90 days		3 (14.3%)	2 (6.7%)	2 (8.3%)	0	*p* = 0.637	*p* = 0.522
Hospital stay (days) ^a^		11.1 (6.01)	10.0 (5.28)	10.1 (5.36)	9.7 (5.50)	*p* = 0.962	*p* = 0.996
ICU or HDU stay, days^a^		1.11 (2.23)	1.5 (2.72)	1.6 (2.82)	1.0 (2.45)	*p* = 0.821	*p* = 0.762
CCI 30 days postoperatively^a^		26.5 (23.38)	28.8 (26.96)	29.6 (26.42)	25.8 (31.37)	*p* = 0.930	*p* = 0.843
*Malign tumor diagnosed (CRC)*		*n* = 42	*n* = 48	*n* = 41	*n* = 7		
Admitted to ICU or HDU postoperatively		6 (14.3%)	5 (10.4%)	2 (4.9%)	3 (42.9%)	*p* = 0.749	*p* = 0.016
Mortality 30 days		4 (9.5%)	1 (12.1%)	1 (2.4%)	0	*p* = 0.181	*p* = 0.465
Mortality 90 days		8 (19.0%)	7 (14.6%)	5 (12.2%)	2 (28.6%)	*p* = 0.778	*p* = 0.589
Hospital stay (days) ^a^		14.5 (19.91)	8.5 (4.25)	7.9 (3.91)	11.7 (4.99)	*p* = 0.013	*p* = 0.005
ICU or HDU stay, days^a^		0.9 (3.60)	0.5 (1.78)	0.2 (0.99)	2.3 (3.68)	*p* = 0.541	*p* = 0.016
CCI 30 days postoperatively^a^		29.4 (20.09)	21.1 (18.98)	17.9 (16.88)	39.1 (21.53)	*p* = 0.020	*p* = 0.007
*Right sided resection*		*n* = 37	*n* = 59	*n* = 45	*n* = 19		
Admitted to ICU or HDU postoperatively		9 (24.3%)	13 (22.0%)	6 (13.3%)	7 (50.0%)	*p* = 0.808	*p* = 0.014
Mortality 30 days		3 (8.1%)	2 (3.4%)	1 (2.2%)	1 (7.1%)	*p* = 0.370	*p* = 0.358
Mortality 90 days		4 (10.8%)	7 (11.9%)	5 (11.1%)	2 (14.3%)	*p* = 1.000	*p* = 1.000
Hospital stay (days) ^a^		8.9 (3.80)	10.2 (8.73)	8.5 (4.59)	15.8 (14.98)	*p* = 0.976	*p* = 0.030
ICU or HDU stay, days^a^		1.2 (2.31)	2.4 (8.02)	0.6 (1.86)	8.1 (15.13)	*p* = 0.862	*p* = 0.010
CCI 30 days postoperatively^a^		34.3 (29.65)	27.9 (28.58)	22.2 (23.59)	46.3 (35.88)	*p* = 0.283	*p* = 0.030
*Left sided resection*		*n* = 61	*n* = 50	*n* = 36	*n* = 14		
Admitted to ICU or HDU postoperatively		16 (26.2%)	14 (28.0%)	9 (25.0%)	5 (35.7%)	*p* = 1.000	*p* = 0.718
Mortality 30 days		4 (6.6%)	3 (6.0%)	2 (5.6%)	1 (7.1%)	*p* = 1.000	*p* = 1.000
Mortality 90 days		6 (9.8%)	7 (14.0%)	3 (8.3%)	4 (28.6%)	*p* = 0.561	*p* = 0.148
Hospital stay (days) ^a^		12.0 (9.23)	10.1 (5.80)	10.0 (6.44)	10.3 (3.91)	*p* = 0.062	*p* = 0.112
ICU or HDU stay, days^a^		1.3 (2.85)	2.0 (4.87)	1.7 (2.85)	2.8 (5.16)	*p* = 0.721	*p* = 0.654
CCI 30 days postoperatively^a^		31.9 (28.17)	35.1 (29.59)	32.2 (29.74)	42.6 (28.87)	*p* = 0.564	*p* = 0.435

^a^Values are: mean (SD).

*Intention-to-treat: conversions are considered as laparoscopic operations.

**As-treated: conversions are considered separately.

**Table 8:** Regression analysis of the Comprehensive Complication Index and 30-day and 90-day mortality

Variable	Exp(B)	95% confidence interval		*P*-value
		Lower	Upper	
*Mortality (30 days)* ^a^				
Surgical technique (Intention-to-treat*)	0.787	0.112	5.531	*p* = 0.810
Surgical technique (As-treated**)	0.376	0.036	3.915	*p* = 0.413
Age	1.049	0.973	1.132	*p* = 0.211
ASA-classification	6.592	1.603	27.102	*p* = 0.009
Severe heart disease	4.374	1.013	18.891	*p* = 0.048
Dementia	0.452	0.078	2.630	*p* = 0.377
Chronic liver disease	3.796	0.246	58.663	*p* = 0.340
Use of anticoagulants	1.221	0.261	5.723	*p* = 0.800
Surgical site contamination	1.391	0.346	5.593	*p* = 0.642
Charlson Comorbidity Index	1.263	0.887	1.798	*p* = 0.195
*Mortality (90 days)* ^a^				
Surgical technique (Intention-to-treat*)	0.632	0.24	1.666	*p* = 0.354
Surgical technique (As-treated**)	1.713	0.876	3.351	*p* = 0.116
Age	1.008	0.963	1.054	*p* = 0.743
ASA-classification	3.039	1.334	6.925	*p* = 0.008
Severe heart disease	1.464	0.47	4.558	*p* = 0.510
Chronic liver disease	10.120	0.715	143.195	*p* = 0.087
Peripheric vascular disease	9.734	1.726	54.893	*p* = 0.010
Use of anticoagulants	2.380	0.703	8.060	*p* = 0.164
Surgical site contamination	1.757	0.672	4.592	*p* = 0.250
Cancer within 5 years or during the surgery	6.567	1.646	26.19	*p* = 0.008
Charlson Comorbidity Index	1.033	0.799	1.334	*p* = 0.805

	B	95% confidence interval		*P*-value
		Lower	Upper	
*Comprehensive Complication Index* ^c^				
Surgical technique (Intention-to-treat*)	− 7.112	− 17.223	2.999	*p* = 0.167
Surgical technique (As-treated**)	− 14.434	− 25.083	− 3.784	*p* = 0.008
Age	0.248	− 0.071	0.567	*p* = 0.127
ASA-classification	12.421	4.021	20.821	*p* = 0.004
Severe heart disease	0.578	− 9.335	10.492	*p* = 0.909
Peripheral vascular disease	14.711	− 1.446	30.867	*p* = 0.074
Dementia	3.123	− 7.762	14.008	*p* = 0.572
Chronic liver disease	32.982	9.005	56.959	*p* = 0.007
Renal disease	12.425	− 4.487	29.338	*p* = 0.149
Use of anticoagulants	8.777	− 2.334	19.888	*p* = 0.121
Admission to ICU or HDU before surgery	22.659	7.543	37.775	*p* = 0.003
Surgical site contamination	8.425	1.385	15.466	*p* = 0.019
Charlson Comorbidity Index	− 0.155	− 1.683	1.372	*p* = 0.841

ICU, Intensive care unit; HDU, High dependency unit

^a^Binary logistic regression analysis

^c^Multiple regression analysis

*Intention-to-treat: conversions are considered as laparoscopic operations

**As-treated: conversions are considered separately

The present study has several strengths and limitations. The main strength lies in its well-planned design, focusing on a specific type of surgery in an emergency setting. Patient records were well-documented, thoroughly evaluated manually, and virtually free of missing data concerning the study variables. Our study featured the longest data collection period (10 years) among the studies reviewed, along with a minimum follow-up of five years to evaluate long-term postoperative survival. The initial patient cohort was sufficiently large to enable robust PSM, resulting in a final sample size adequate to minimize the effect of confounding variables and selection bias. The 1:1 patient ratio for the groups was chosen as retaining larger cohort of open surgery patients could have caused higher bias on otherwise heterogenous initial patient population. The diversity of the cohort may reduce the direct comparability of our findings with studies restricted to specific patient groups, such as those undergoing surgery for colorectal cancer. Furthermore, the inclusion of multiple surgical indications may obscure indication-specific effects and limit the ability to identify differences attributable to individual underlying pathologies. Conversely, limiting the analysis exclusively to cancer patients would exclude a substantial proportion of individuals undergoing emergency colorectal resection and, therefore, diminish the generalizability of the results. The primary limitation is the non-randomized nature of patient selection and surgical approach, which was determined by the operating surgeon’s preference and the clinical circumstances at the time. Despite PSM, some differences in baseline characteristics persisted between the groups, particularly in terms of the severity of surgical site contamination. The decision not to include this variable on PSM grouping analysis was the fact that the severity of contamination was an intraoperative finding, while the matching was conducted based on data prior to the surgery, and these preoperative factors already included the diagnosis of perforation based on clinical and radiographic findings.

Consistent with findings from elective surgery, laparoscopy appears to offer advantages in terms of postoperative recovery and a reduced burden of complications in patients undergoing emergency surgery for bowel obstruction without perforation and in those diagnosed with colorectal cancer. For other surgical indications, outcomes were generally comparable between the approaches. However, given the limited evidence currently available and the inherent susceptibility of retrospective studies to selection bias, adequately powered and well-designed randomized controlled trials are warranted to validate these findings.

## Supplementary Information

Below is the link to the electronic supplementary material.

Supplementary Material 1

## Data Availability

No datasets were generated or analysed during the current study.
