# Supplementary material for: Laparoscopic approach for emergency colon resection: a retrospective propensity score-matched study
Source: World J Emerg Surg. 2026 Aug 10;21:63. doi: 10.1186/s13017-026-00721-y (PMC13746622; doi:10.1186/s13017-026-00721-y)
Supplement: Supplementary file 1 [file 13017_2026_721_MOESM1_ESM.docx]

| Supplementary table 1. Propensity score matching covariates before and after the analysis. | | | | | | | |
| --- | --- | --- | --- | --- | --- | --- | --- |
|  |  |  |  |  |  |  |  |
|  |  | **Original** | |  | **Matched** | |  |
|  |  | Open | Laparoscopy ITT | SMD | Open | Laparoscopy ITT | SMD |
|  |  | n=368 | n=116 |  | n=116 | n=116 |  |
|  |  |  |  |  |  |  |  |
| Age^a^ | | 69.2 (14.63) | 69.3 (14.29) | 0.023 | 68.3 (15.31) | 69.3 (14.29) | 0.025 |
| Body mass index^a^ | | 25.6 (4.53) | 25.5 (4.66) | 0.033 | 25.5 (4.30) | 25.5 (4.66) | 0.016 |
| ASA grade | |  |  |  |  |  |  |
|  | I | 9 (2.4%) | 4 (3.4%) | 0.060 | 1 (0.9%) | 4 (3.4%) | 0.180 |
|  | II | 79 (21.5%) | 32 (27.6%) | 0.142 | 34 (29.3%) | 32 (27.6%) | 0.038 |
|  | III | 145 (39.4%) | 63 (54.3%) | 0.300 | 51 (44.0%) | 63 (54.3%) | 0.206 |
|  | IV | 113 (30.7%) | 16 (13.8%) | 0.398 | 27 (23.3%) | 16 (13.8%) | 0.246 |
|  | V | 22 (6.0%) | 1 (0.9%) | 0.305 | 3 (2.6%) | 1 (0.9%) | 0.134 |
| Cancer within 5 years | |  |  |  |  |  |  |
|  | None | 204 (55.4%) | 57 (49.1%) | 0.127 | 64 (55.2%) | 57 (49.1%) | 0.123 |
|  | Local | 105 (28.5%) | 39 (33.6%) | 0.110 | 37 (31.9%) | 39 (33.6%) | 0.036 |
|  | Metastatic | 59 (15.8%) | 20 (17.2%) | 0.038 | 15 (12.9%) | 20 (17.2%) | 0.121 |
| Cerebrovascular accident | |  |  |  |  |  |  |
| or TIA | | 36 (9.8%) | 13 (11.2%) | 0.046 | 14 (12.1%) | 13 (11.2%) | 0.028 |
| Severe heart disease | | 77 (20.9%) | 20 (17.2%) | 0.094 | 22 (19.0%) | 20 (17.2%) | 0.047 |
| Peripheral vascular disease | | 19 (5.2%) | 7 (6.0%) | 0.035 | 4 (3.4%) | 7 (6.0%) | 0.124 |
| Dementia | | 48 (13.0%) | 8 (6.9%) | 0.206 | 19 (16.4%) | 8 (6.9%) | 0.302 |
| Diabetes mellitus | | 44 (12.0%) | 15 (12.9%) | 0.027 | 11 (9.5%) | 15 (12.9%) | 0.108 |
| Chronic liver disease | | 9 (2.4%) | 2 (1.7%) | 0.050 | 3 (2.6%) | 2 (1.7%) | 0.062 |
| Chronic kidney disease | | 19 (5.2%) | 4 (3.4%) | 0.089 | 6 (5.2%) | 4 (3.4%) | 0.089 |
| Use of anticoagulants | | 55 (14.9%) | 15 (12.9%) | 0.057 | 14 (12.1%) | 15 (12.9%) | 0.024 |
| Use of oral corticosteroids | | 30 (8.2%) | 5 (4.3%) | 0.163 | 6 (5.2%) | 5 (4.3%) | 0.042 |
| Admittance to ICU or HDU before surgery | | 29 (7.9%) | 8 (6.9%) | 0.038 | 5 (4.3%) | 8 (6.9%) | 0.114 |
| Reason for surgery | |  |  |  |  |  |  |
|  | Malignant tumor (CRC) | 124 (33.7%) | 48 (41.4%) | 0.159 | 42 (36.2%) | 48 (41.4%) | 0.107 |
|  | Malignant tumor (not CRC) | 14 (3.8%) | 3 (2.6%) | 0.069 | 3 (2.6%) | 3 (2.6%) | 0.000 |
|  | Benign obstruction | 78 (21.2%) | 19 (16.4%) | 0.123 | 28 (24.1%) | 19 (16.4%) | 0.192 |
|  | Ischemia | 29 (7.9%) | 6 (5.2%) | 0.110 | 4 (3.4%) | 6 (5.2%) | 0.089 |
|  | Diverticulitis | 98 (26.6%) | 29 (25.0%) | 0.004 | 33 (28.4%) | 29 (25.0%) | 0.077 |
|  | Colitis / IBD | 12 (3.3%) | 4 (3.4%) | 0.006 | 2 (1.7%) | 4 (3.4%) | 0.109 |
|  | Other | 7 (1.9%) | 7 (6.0%) | 0.218 | 4 (3.4%) | 7 (6.0%) | 0.124 |
| Emergency surgery indication | |  |  |  |  |  |  |
|  | Obstruction | 153 (41.6%) | 57 (49.1%) | 0.151 | 55 (47.4%) | 57 (49.1%) | 0.034 |
|  | Perforation | 141 (38.3%) | 29 (25.0%) | 0.287 | 40 (34.5%) | 29 (25.0%) | 0.208 |
|  | Other | 74 (20.1%) | 30 (25.9%) | 0.138 | 21 (18.1%) | 30 (25.9%) | 0.189 |
|  |  |  |  |  |  |  |  |

TIA, transient ischemic attack; IBD, inflammatory bowel disease; ICU, intensive care unit; HDU, high dependency unit; CRC, colorectal cancer; ITT, intention-to-treat.

^a^Values are: mean (SD).

| Supplementary table 2. Reasons for conversions and regression analysis for the risk factors for conversion. | | | | |  | |  | |
| --- | --- | --- | --- | --- | --- | --- | --- | --- |
| **Reason for conversion** | n=116 | | | |  | |  | |
| No conversion | 85 (73.3%) | | | | |  |  |  |
| Insufficient working space | 12 (10.3%) | | | | |  |  |  |
| Abdominal adhesions | 2 (1.7%) | | | | |  |  |  |
| Unclear anatomy | 3 (2.6%) | | | | |  |  |  |
| Surgery not technically possible | 13 (11.2%) | | | | |  |  |  |
| Other | 1 (0.9%) | | | | |  |  |  |
|  |  |  | |  | | | |  |
|  |  |  | |  | | | |  |
| Variable | B | 95% confidence interval | | P-value | | | |  |
|  |  | Lower | Upper |  | | | |  |
| **Conversion** |  |  |  |  | | | |  |
|  |  |  |  |  | | | |  |
| Chronic liver disease | 0.689 | 0.078 | 1.299 | p=0.027 | | | |  |
| Emergency surgery indication |  |  |  |  | | | |  |
| Obstruction | -0.010 | -0.217 | 0.198 | p=0.925 | | | |  |
| Perforation | 0.216 | -0.020 | 0.452 | p=0.072 | | | |  |
| Other | -0.042 | -0.430 | 0.347 | p=0.832 | | | |  |

| Supplementary table 3. Kaplan-Meier analysis for long-term mortality. | | | | | |
| --- | --- | --- | --- | --- | --- |
|  |  | Estimate | Std. Error | Mean (95% CI) | p-value |
|  |  |  |  |  | n=116 |
|  |  |  |  |  |  |
| **A) Survival functions for all patients** | | | | | |
| Laparoscopy ITT | | 6.111 | 0.512 | 5.109-7.114 | p=0.466 |
| Open | | 7.018 | 0.539 | 5.962-8.073 |  |
|  | |  |  |  |  |
| **B) Survival functions for all patients, conversions as a separate group** | | | | | |
| Laparoscopy AT | | 6.341 | 0.589 | 5.185-7.496 | p=0.456 |
| Open | | 7.018 | 0.539 | 5.962-8.073 |  |
| Conversion | | 5.423 | 0.984 | 3.494-7.351 |  |
|  | |  |  |  |  |
